# Supplementary material for: Resolving the full spectrum of human genome variation using Linked-Reads
Source: Genome Res. 2019 Apr;29(4):635–45. doi: 10.1101/gr.234443.118 (PMC6442396; doi:10.1101/gr.234443.118)
Supplement: Supplemental Material [file supp_gr.234443.118_Supplemental_longranger-2.2.zip › longranger-2.2.tar.bz2!/lr2.2-public/lib/stan/cmdstan/stan/doc/api/html/meta_2value__type_8hpp_source.html]

Stan: src/stan/math/meta/value\_type.hpp Source File


|  |  |
| --- | --- |
|  | Stan  2.5.0  probability, sampling & optimization |


- Main Page
- Related Pages
- Namespaces
- Classes
- Files

- File List
- File Members

All Classes Namespaces Files Functions Variables Typedefs Enumerations Enumerator Friends Macros Pages

- src
- stan
- math
- meta

value\_type.hpp

Go to the documentation of this file.

1 #ifndef STAN\_\_MATH\_\_META\_\_VALUE\_TYPE\_HPP

2 #define STAN\_\_MATH\_\_META\_\_VALUE\_TYPE\_HPP

3

4 #include <vector>

5

6 namespace stan {

7

8  namespace math {

9

20  template <typename T>

21  struct value\_type {

22  };

23

24

31  template <typename T>

32  struct value\_type<const T> {

33  typedef typename value\_type<T>::type type;

34  };

35

36

43  template <typename T>

44  struct value\_type<std::vector<T> > {

45

50  typedef typename std::vector<T>::value\_type type;

51

52  };

53

54

55  }

56 }

57

58

59 #endif

stan::math::value\_type< const T >::type

value\_type< T >::type type

**Definition:** value\_type.hpp:33

stan::math::value\_type< std::vector< T > >::type

std::vector< T >::value\_type type

Type of value stored in a standard vector with type T entries.

**Definition:** value\_type.hpp:50

stan::math::value\_type

Primary template class for metaprogram to compute the type of values stored in a container.

**Definition:** value\_type.hpp:21


---

[ Stan Home Page ]

*© 2011–2014,
Stan Development Team.*
